# Current use of complementary and conventional medicine for treatment of pediatric patients with gastrointestinal disorders

**DOI:** 10.3389/fphar.2023.1051442

**Published:** 2023-01-27

**Authors:** Casey L. Sayre, Venkata Kashyap Yellepeddi, Kathleen M. Job, Lubov V. Krepkova, Catherine M. T. Sherwin, Elena Y. Enioutina

**Affiliations:** ^1^ Division of Clinical Pharmacology, Pediatrics, School of Medicine, Salt Lake City, UT, United States; ^2^ College of Pharmacy, Roseman University of Health Sciences, South Jordan, UT, United States; ^3^ Center of Medicine, All-Russian Research Institute of Medicinal and Aromatic Plants (VILAR), Moscow, Russia; ^4^ Department of Pediatrics, Boonshoft School of Medicine, Wright State University, Dayton, OH, United States

**Keywords:** gastrointestinal disease, pediatric patients, conventional treatment, complementary treatment, dietary supplements, probiotics

## Abstract

Infants, children, and adolescents are at risk of experiencing a multitude of gastrointestinal disorders (GID). These disorders can adversely affect the quality of life or be life-threatening. Various interventions that span the conventional and complementary therapeutic categories have been developed. Nowadays, parents increasingly seek complementary options for their children to use concurrently with conventional therapies. Due to the high prevalence and morbidity of diarrhea, constipation, and irritable bowel syndrome (IBS) in children, in this review, we decided to focus on the current state of the evidence for conventional and complementary therapies used for the treatment of these diseases in children. Diarrhea treatment focuses on the identification of the cause and fluid management. Oral rehydration with supplementation of deficient micronutrients, especially zinc, is well established and recommended. Some probiotic strains have shown promise in reducing the duration of diarrhea. For the management of constipation, available clinical trials are insufficient for conclusive recommendations of dietary modifications, including increased use of fruit juice, fiber, and fluid. However, the role of laxatives as conventional treatment is becoming more established. Polyethylene glycol is the most studied, with lactulose, milk of magnesia, mineral oil, bisacodyl, and senna presenting as viable alternatives. Conventional treatments of the abdominal pain associated with IBS are poorly studied in children. Available studies investigating the effectiveness of antidepressants on abdominal pain in children with IBS were inconclusive. At the same time, probiotics and peppermint oil have a fair record of benefits and safety. The overall body of evidence indicates that a careful balance of conventional and complementary treatment strategies may be required to manage gastrointestinal conditions in children.

## 1 Introduction

Gastrointestinal disorders (GID) are common conditions in children and a significant source of distress for parents and children. The symptoms of gastrointestinal disorders can severely limit a child’s quality of life. Beyond that, some GID can result in children’s mortality; for example, an estimated 5,00,000 children under 5 years old die worldwide each year from the devastating effects of persistent diarrhea (Shane et al., 2017; Guarino et al., 2018; Szajewska et al., 2020b). Constipation is another condition that affects many children, ranging in prevalence in this population between 0.5%–32.2% (Koppen et al., 2018). Additionally, irritable bowel syndrome (IBS) and its symptoms affect an estimated 6%–14% of children and 22%–35.5% of adolescents (Hyams et al., 1996; Lake, 1999; Miele et al., 2004; Devanarayana and Rajindrajith, 2018). Due to the high prevalence and morbidity of these conditions in children, it is essential to understand the effectiveness of the available treatments for the three common pediatric gastrointestinal conditions: diarrhea, constipation, and irritable bowel syndrome (IBS). These three GIDs are the focus of this review since these diseases can lead to a child’s morbidity or even mortality and decrease a child’s quality of life. Additionally, many parents of children with GID must confront how to best treat their children. This can be daunting with the multitude of conventional and complementary self-care products available today. Complementary products are meant to be used alongside conventional treatments and may include herbal dietary supplements or probiotics. About 60% of parents of children with GID have sought complementary treatments, and more than 90% believed it was important for pediatricians to be fluent in the latest research on complementary treatments for GID in children (Vlieger et al., 2008). However, research into complementary treatments for children is limited compared to similar studies in adults, creating a need for additional rigorous scientific exploration of this topic (Vlieger et al., 2008; Anheyer et al., 2017).

Despite the preference for complementary therapies among parents seeking to treat their children for GID (Vlieger et al., 2008), care should be taken to avoid unsafe and unproven interventions. Although several complementary treatments show preliminarily encouraging results, there is a relative lack of large-sample data in some cases, implying the need to continue researching these treatments. Weaknesses in methodology, small sample size, and variability in the definition of outcomes are just a few common weaknesses in clinical studies of complementary therapies for GID in children. These flaws limit the applicability of the study findings and make requisite the competent clinical evaluation of an individual child.

## 2 Methods

A comprehensive evaluation of the existing literature was conducted through searches in electronic databases, including PubMed, Embase, Scopus, ScienceDirect, and Web of Science, for literature published between January 1960 and May 2022. Searches were conducted using the keywords: irritable bowel syndrome, diarrhea, constipation, pediatric, complementary medicine, herbal remedies, and dietary supplements. In this review, diarrhea, constipation, and then irritable bowel syndrome will be presented. In each section, background on the GID is first provided, followed by a summary of the conventional treatment. Finally, an exploration of the currently available complementary therapies will be discussed.

## 3 Diarrhea

Changes in stool consistency and weight are the primary characteristics by which diarrhea is defined in children. Soft to watery stool consistency is typical, with an increased weight of greater than 250 g per day in children weighing more than 10 kg. For example, in children weighing less than 10 kg, a stool weight greater than 20 g per kilogram per day indicates diarrhea (Schiller et al., 2017). In terms of incidence, a large study reported that between 3% and 20% of children under 5 years of age experienced an episode of diarrhea worldwide (Walker et al., 2013). Geographic and socioeconomic differences in incidence appear as diarrheal symptoms persist into the chronic range, with chronic diarrhea being more common in underdeveloped or developing countries (Vernacchio et al., 2006; Walker et al., 2013).

Acute gastroenteritis, commonly called “acute infectious diarrhea,” is a common infectious disease in children, with 1.3 million deaths worldwide annually and about 500,000 deaths in children under 5 years old (Shane et al., 2017; Guarino et al., 2018; Szajewska et al., 2020b). Acute gastroenteritis also results in numerous outpatient or emergency department visits and hospitalizations. The pathophysiology of acute gastroenteritis involves inflammation of the gastrointestinal tract and is commonly caused by bacterial, viral, or parasitic pathogens. The typical microorganisms responsible for bacterial gastroenteritis include *Escherichia coli, Salmonella spp., Shigella spp., Vibrio parahaemolyticus, Staphylococcus aureus, Clostridium perfringens, Bacillus cereus, Listeria monocytogenes, Yersinia enterocolitica, and Campylobacter jejuni* (Ryoo, 2021)*.* The viruses identified as causing acute gastroenteritis are norovirus, rotavirus, human adenovirus, human astrovirus, and sapovirus (Ryoo, 2021). Finally, the four protozoa types linked to acute gastroenteritis are *Cryptosporidium parvum, Giardia lamblia, Entamoeba histolytica, and Cyclospora cayetanensis* (Ryoo, 2021).

Symptoms of diarrhea that persist for more than 4 weeks are categorized as chronic (Schiller et al., 2017). Although chronic diarrhea prevalence is greater in children from underdeveloped countries (1.4%–28.4%), it is estimated that only 10% of acute episodes transition to chronic symptoms of diarrhea (Vernacchio et al., 2006; Mathai et al., 2011). In addition to unpleasant diarrheal symptoms to the child, chronic diarrhea is a significant cause of mortality in countries with less developed economies and little money spent on healthcare (Mathai et al., 2011).

### 3.1 Conventional treatment of diarrhea in children

The US Centers for Disease Control and Prevention (CDC), the World Health Organization (WHO), and the American Academy of Pediatrics (AAP) have recommended oral rehydration therapy (ORT) as the mainstay therapy to treat acute diarrhea in children (King et al., 2003; World Health, 2004; Carson et al., 2016) (Table 1). The ORT treatment reduces the need for intravenous fluid therapy and can reduce the duration of hospital stays. However, vomiting in children with acute gastroenteritis can impede the ORT and is often treated with antiemetics such as ondansetron. The Infectious Disease Society of America (IDSA) recommends against empiric antimicrobial therapy for patients who are not immunocompromised and without recent international travel (Shane et al., 2017).

**TABLE 1:** Conventional treatments for chronic diarrhea in children.

Name of the drug/treatment	Mechanism of action	Effectiveness/adverse events
Oral rehydration therapy (ORT)	Fluid replacement	Reduced the need for IV fluid therapy; Reduced the duration of hospital stay (King et al., 2003; World Health, 2004; Ryoo, 2021)
Pancreatic enzymes	Replacement of deficiency	Near complete resolution of fat malabsorption-related diarrhea (Sankararaman et al., 2019)
Loperamide	Anti-motility agent	Helpful in fluid management of non-infectious chronic diarrhea, it can slow the excretion of offending microbes in infectious diarrhea (DuPont and Hornick, 1973; Kellermayer and Shulman, 2021)
Diphenoxylate-atropine	Anti-motility agent	Helpful in fluid management of non-infectious chronic diarrhea, it can slow the excretion of offending microbes in infectious diarrhea (DuPont and Hornick, 1973; Kellermayer and Shulman, 2021)
Somatostatin	Endocrine-metabolic agent	Relieves symptoms in chronic diarrhea caused by chemotherapy, neuroendocrine tumors, microvillous inclusion disease, or enterotoxins (Bisset et al., 1993; Pai et al., 2011)
Octreotide	Endocrine-metabolic agent	Relieves symptoms of chronic diarrhea caused by chemotherapy, neuroendocrine tumors, microvillous inclusion disease, or enterotoxins (Bisset et al., 1993; Pai et al., 2011). Decreased stool weight in children with cryptosporidiosis (Guarino et al., 1998)

Treatment of chronic diarrhea in children should consider the underlying cause and geographic and socioeconomic considerations. For example, in developed countries, causes of chronic diarrhea can range from dietary factors, developmental, and malabsorption disorders, immune disorders, or infectious agents (Bhutta et al., 2004; Binder, 2006). This is in contrast to the typical causes of chronic diarrhea in underdeveloped countries, which are either poor nutrition or recurrent gastrointestinal infections (Gibbons and Fuchs, 2007).

Malabsorption and malnutrition causes are serious in infants. Treatment focuses on establishing proper nutrition and may necessitate enteral feeding. Supplementation of deficient micronutrients is also a cornerstone of treatment, as deficiencies of selenium, folic acid, vitamin A, copper, and zinc may be seen in children with malnutrition causing chronic diarrhea (Bhan et al., 2003). In addition, zinc is at particular risk of significant loss from diarrhea, and supplementation is recommended and discussed further in the complementary treatment section.

Fat malabsorption due to pancreatic insufficiency can cause chronic diarrhea in children (Sankararaman et al., 2019). Historically, this condition was managed by restricting the amount of fat in the diet, often worsening the symptoms of malnutrition (Sankararaman et al., 2019). The development of pancreatic enzyme replacement therapy with orally delivered pancreatic enzymes has allowed the near complete correction of the enzyme deficiency in fat digestion (Sankararaman et al., 2019) (Table 1). Lactose intolerance or malabsorption, as well as food allergies and sensitivities, are some examples of dietary causes of chronic diarrhea. Isolating and removing the suspected offending dietary component from the diet for seven to 10 days is suggested (Kellermayer and Shulman, 2021).

Anti-diarrheal drugs are not typically recommended for use in children with diarrhea. However, anti-motility agents like loperamide and diphenoxylate-atropine can be helpful for fluid management in chronic diarrhea where the cause has been determined not to be infectious (Kellermayer and Shulman, 2021). These drugs are not recommended in chronic diarrhea due to enteric infection as decreasing intestinal motility with diphenoxylate-atropine, for example, has the potential adverse effect of delaying the excretion of the offending organisms (DuPont and Hornick, 1973). Some other conventional drugs have a role in treating chronic diarrhea with specific causes. For example, in chronic diarrhea caused by chemotherapy, neuroendocrine tumors, microvillous inclusion disease, or enterotoxins, somatostatin or octreotide have shown benefits (Bisset et al., 1993; Pai et al., 2011) (Table 1). Further, octreotide could decrease stool weight in children with cryptosporidiosis (Guarino et al., 1998).

### 3.2 Complementary treatment of diarrhea in children

**FIGURE 1:**
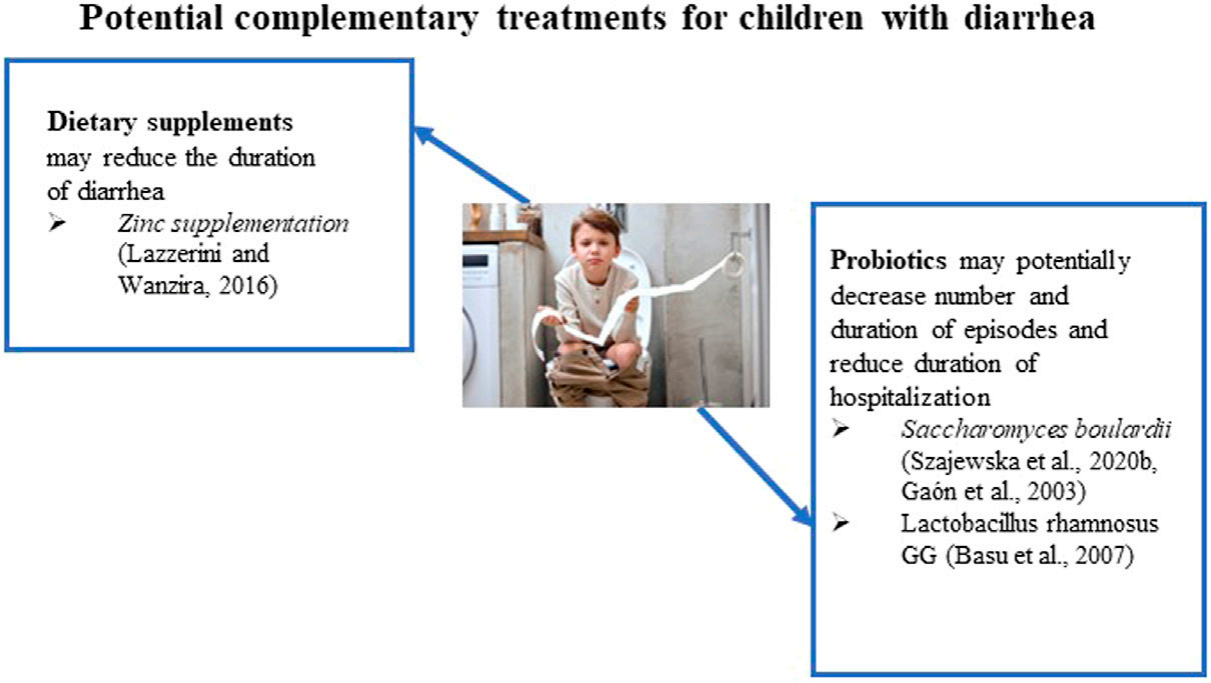
Potential complementary treatments for children with diarrhea.

In addition to ORT, IDSA recommends ancillary management options, including probiotics and oral zinc supplementation (Shane et al., 2017) (Figure 1). Probiotics in trials reduced the diarrhea duration and stool frequency with a sustained beneficial effect across all outcomes (Shane et al., 2010; Shane et al., 2017). Oral zinc supplementation reduces the duration of diarrhea in children 6 months to 5 years of age who reside in countries with a high prevalence of zinc deficiency or malnourished children.

Probiotics may have multiple mechanisms to account for their effectiveness in diarrhea. These mechanisms are associated with an improvement of the immune response to an intestinal pathogen, including increased production of mucin and antimicrobial peptides and improved cell junction stability (do Carmo et al., 2018). The most recommended probiotics for the treatment of acute gastroenteritis are *Saccharomyces boulardii*) *Lactobacillus rhamnosus GG*)*, Lactobacillus helveticus, and Lactobacillus reuteri* (Szajewska et al., 2020a)*.* The European Society for Paediatric Gastroenterology, Hepatology, and Nutrition Working Group on Probiotics and Prebiotics (ESPGHAN-WG) updated their recommendations in 2020 on the use of probiotics in children with acute gastroenteritis (Szajewska et al., 2020a). The ESPGHAN-WG made weak recommendations for *S. boulardii* (low to extremely low certainty of the evidence); *L. rhamnosus GG* (extremely low certainty of the evidence); *L. reuteri* DSM 17938 (low to extremely low certainty of the evidence); and L. *rhamnosus* 19,070-2 and *L. reuteri* DSM 12246 (extremely low certainty of the evidence). The WG made a strong recommendation against *L. helveticus* R0052 and *L. rhamnosus* R0011 (moderate certainty of the evidence) and a weak recommendation against *Bacillus clausii* strains O/C, SIN, N/R, and T (very low certainty of the evidence) (Szajewska et al., 2020a).

*S. boulardii* is a widely available probiotic yeast and is the most investigated probiotic for treating acute gastroenteritis in children (Szajewska et al., 2020b). Several randomized clinical trials have shown that *S. boulardii* is moderately effective in treating children with acute gastroenteritis by shortening the duration of diarrhea. The possible mechanisms for the activity of *S. boulardii* include interference with the attachment of causative pathogens, toxin inactivation (e.g., *C. difficile* toxins), normalization of the transcellular transport of chloride and reduced fluid loss, and immunomodulatory effects (Szajewska et al., 2020b). The researchers reported the results of a systematic review of available data and meta-analysis of *S. boulardii* for treatment of acute gastroenteritis in children and included 29 randomized controlled trials (RCTs) in their analysis (Szajewska et al., 2020b). The results have shown that *S. boulardii* use reduced the duration of diarrhea and hospitalization. However, the statistical calculations from the meta-analysis showed very low-quality evidence of *S. boulardii*’*s* activity.

Several trials have shown modest benefits in chronic diarrhea with probiotic administration. The efficacy of *S. boulardii* and *Lactobacillus spp.* was studied in a double-blind, placebo-controlled trial (Gaón et al., 2003). The probiotic cohort showed significant decreases in the duration and number of diarrhea episodes. Another randomized, controlled, double-blinded study reviewed the effect of *Lactobacillus rhamnosus* GG on 117 participants with persistent diarrhea (Basu et al., 2007). A significant reduction in diarrhea duration and frequency was achieved. No adverse effects of *Lactobacillus rhamnosus* GG were reported in the study. Additionally, a lower duration of hospital stay was observed. However, the current evidence is insufficient to make a confident recommendation to treat chronic or persistent diarrhea with probiotics (Bernaola Aponte et al., 2013). The authors note this is primarily due to the small number of studies and a small number of participants enrolled in the studies.

As mentioned previously, the IDSA and WHO recommend 20 mg of oral zinc supplementation for 10–14 days in addition to ORT (World Health, 2004; Shane et al., 2017). The action of zinc in reducing acute gastroenteritis in children involves modulating the cell membrane and cellular function of immune cells, thereby improving immunity (World Health, 2004). It has been also reported that the mechanism of action of zinc in diarrhea involves multiple pathways, including regulation of fluid transport in the intestines as well as modification of the intestinal mucosa (Berni Canani et al., 2011). In a meta-analysis of 24 RCTs, oral zinc supplementation shortened the duration of acute diarrhea in children 6 months to 5 years of age by 10 h (Lazzerini and Wanzira, 2016). Furthermore, in children who have signs of malnutrition, a greater reduction of 27 h in the duration of diarrhea was observed (Lazzerini and Wanzira, 2016). A group of investigators reported an extensive meta-analysis that included studies in Chinese literature evaluating zinc’s effectiveness in treating diarrhea in children (Lamberti et al., 2013). They identified 89 Chinese and 15 non-Chinese studies for the review, including studies in ten countries from all WHO geographic regions, and analyzed a total of 18,822 diarrhea cases (9,469 patients received zinc and 9,353 patients were used as a control). The pooled data yielded an overall 26% (95% CI: 20%-32%) reduction in the estimated relative risk of diarrhea lasting beyond 3 days among zinc-treated children (Lamberti et al., 2013).

In addition to probiotics and zinc supplementation, herbal medicines have a history of treating diarrhea in children (Shane et al., 2017). In Africa, some commonly used herbs for the treatment of acute gastroenteritis include extracts of the neem tree (*A. indica*) and *E. africana* (Adeyemi et al., 2021). A commonly used herbal treatment in China is the Gengen Huangqin Huanglian Decoction (GHHD) (Wu et al., 2020). This Traditional Chinese Medicine based product is composed of *Puerariae lobatae radix* (Gegen), *Scutellariae radix* (Huangqin), *Coptidis rhizoma* (Huanglian), *glycyrrhiza* (Gancao) (Wu et al., 2020). Clinical trial evidence for these herbal medicines as a complementary treatment for diarrhea in children has not been published.

## 4 Constipation

The normal defecation patterns in children can vary widely. By 1–4 years of age, most children have stool every or every other day (Noe, 2018). Constipation is a common condition in children, with the majority demonstrating no underlying medical reason (Kennedy, 2011). This predominant form of constipation is termed functional constipation. Genetic, metabolic, hormonal, abnormal anatomy, and psychological causes may also lead to the development of constipation in children. Additionally, certain medications (e.g., opioids) can contribute to the development of temporal constipation in children. The prevalence rate of constipation in children ranges between 0.5–32.2% (van den Berg et al., 2006; Koppen et al., 2018).

Constipation in children can be characterized by the absence of a bowel movement for a few days, passing hard, dry stools, straining, pain while defecating, bloating, and even not feeling hungry (Koppen and Benninga, 2022). A useful diagnostic tool to identify constipation in children could be a frequency of defecation of ≤2 times a week and a pellet-like, dry stool (Ho and How, 2020; Koppen and Benninga, 2022). When symptoms of constipation are recent in onset and last for less than 8 weeks, it is considered acute (Sood, 2021b). When these symptoms last over 3 months, a child is considered to have chronic constipation. Signs useful in identifying chronic constipation in children include weight loss or poor weight gain, constipation from birth or early infancy, urinary incontinence, delayed growth, and a family history of Hirschsprung disease (Noe, 2018). Most cases of chronic constipation tend to be functional (Loening-Baucke, 2005; Koppen and Benninga, 2022). Constipation can become chronic due to the prolonged presence of impacted stool in the colon. This can cause the colon to become distended and unresponsive to normal stool burden (Sood, 2021a). When symptoms occur in infants less than one-month-old, strong suspicion should be given to a potential organic cause of the symptoms (Tabbers et al., 2014).

### 4.1 Conventional treatment of constipation in children

In the first year of life, infants typically transition from a diet of breast milk to formula or subsequently a liquid diet to a solid diet, which may include the introduction of cow’s milk. These transitions can often coincide with inadequate amounts of dietary fiber and fluid, potentially resulting in constipation. Dietary modifications are often the first recommendation in infants with acute constipation (Sood, 2021b). If dietary changes do not resolve the symptoms in infants on a liquid diet, lactulose may be added to the formula (Tabbers et al., 2010; Tabbers et al., 2014; Sood, 2021b). However, further evaluation by a healthcare provider is necessary if symptoms do not resolve. For hard and impacted stools, glycerin suppositories or rectal thermometer stimulation of the rectum may be useful (Zenk et al., 1993; Tabbers et al., 2014). Though, repeated use of glycerin suppositories should be avoided as the infant can become trained to this stimulation to defecate (Abi-Hanna and Lake, 1998). Additionally, glycerin is a potential irritant to the rectal mucosa and anus.

Conventional treatment for acute constipation in children older than 1 year highly depends on whether these children have pain following defecation, bleeding, or anal fissures. Children with constipation could also acquire withholding behavior. These children may be started on polyethylene glycol (PEG) with or without electrolytes (Table 2). The 0.4 g/kg/day dose of PEG was recommended by several authors (Pashankar and Bishop, 2001; Tabbers et al., 2014). In cases of high fecal impaction, the PEG dose can be increased to 1.0–1.5 g/kg/day. If PEG is not tolerated or unavailable, lactulose or magnesia milk may be utilized (Loening-Baucke and Pashankar, 2006; Ratanamongkol et al., 2010; Tabbers et al., 2014).

**TABLE 2:** Conventional treatments for constipation in children.

Name of the drug/treatment	Mechanism of action	Effectiveness/adverse events
Lactulose	Osmotic laxative	Improved stool consistency, safe in all ages (Tabbers et al., 2010; Tabbers et al., 2014)
Glycerin suppositories	Osmotic laxative	Softened stool consistency (Zenk et al., 1993)
Polyethylene glycol (PEG)	Osmotic laxative	Improved stool consistency and increased defecation frequency (Pashankar and Bishop, 2001; Tabbers et al., 2014) (Youssef et al., 2002; Loening-Baucke et al., 2004; Michail et al., 2004; Gordon et al., 2016; Bekkali et al., 2018)
Milk of magnesia	Osmotic laxative	Improved stool consistency and increased defecation frequency (Loening-Baucke and Pashankar, 2006; Ratanamongkol et al., 2010)
Senna	Stimulant laxative	Generally well tolerated, some perianal blisters after nigh time accidents (Vilanova-Sanchez et al., 2018)
Bisacodyl	Stimulant laxative	Effective and safe to use in children for an extended period (Bonilla et al., 2020)

If pain following defecation, bleeding, or anal fissures are absent in children with acute constipation, the initial approach can be to educate parents on toileting and dietary changes, which could lead to non-pharmacologic improvement of constipation in their children (Sood, 2021b; Koppen and Benninga, 2022). Emphasis on adequate dietary fiber and fluid intake should be stressed to prevent relapse of symptoms.

If functional cases of chronic constipation in infants are confirmed, non-digestible osmotically active fibers present in pear, apple, and prune juices often become the first intervention tested (Sood, 2021a). Interestingly, as juices containing non-digestible osmotically active fibers are common foods in the diet of infants, they have been used as non-pharmacologic first-line interventions in infants with chronic constipation. Dark corn syrup was also recommended interchangeably with the fruit juices mentioned above juices. However, it is no longer used due to the variability in the presence of osmotically active carbohydrates (Sood, 2021a).

Pharmacotherapy may be considered in infants if osmotic non-digestible fiber and juices’ carbohydrate symptoms fail to improve infants’ signs and symptoms of chronic constipation (Table 2). Lactulose, an osmotic laxative, is commonly used with success at 1 mL/kg once or twice daily (Sood, 2021a). Another pharmaceutical agent that is gaining popularity in infants with chronic constipation is PEG has been used successfully in adults, but its safety and efficacy in infants and children are not well established (Loening-Baucke et al., 2004; Michail et al., 2004; Bekkali et al., 2018). In several studies, a dose of 0.8 g/kg was well tolerated and produced a good response (Sood, 2021a). It has been reported that using PEG 4000 at a dose of 0.72 g/kg/day for more than 6 months was safe and did not lead to significant adverse reactions (Bae, 2010). However, transient hyperphosphatemia was noticed in several children. Glycerin suppositories help achieve the removal of fecal impaction, a desirable outcome for chronic constipation; however, their use should remain occasional due to the risk of increased anal irritation and the worsening of constipation symptoms (Sood, 2021a).

Children with severe impaction may require rectally administered medications to achieve removal of fecal impaction. The rectal route of administration is reserved for severe cases as it is invasive and difficult to do in some children. Sodium phosphate, saline, and mineral oil enemas have all been used for this purpose (Sood, 2021a).

Immediately after the successful removal of fecal impaction, a pharmacological maintenance regimen should be initiated. This is important in the retraining of the colon and the avoidance of impaction recurrence. Titration of a daily dose of osmotic laxative and a stimulant laxative should be made if needed to achieve passage of soft stool once per day (Hyams et al., 2016).

Additional pharmacological treatment with herbal laxatives may be used. For example, Senna, a common name for fruits and/or leaves of the plant *Senna alexandrina,* is an FDA-approved non-prescription laxative. The sennosides present in this plant irritates bowel epithelial lining resulting in the laxative effect. The FDA has approved it only for short-term use. However, the safety of this drug has been poorly studied in infants and children and therefore requires careful consideration when recommended to infants or children with chronic constipation. In addition, long-term high-dose senna use may result in blisters after defecation and/or prolonged contact of irritant with mucosa and skin of a child (Vilanova-Sanchez et al., 2018).

The treatment strategy for chronic constipation in older children includes establishing normal levels of liquid and fiber intake and physical activity. Pharmacological interventions address incidents of fecal impaction and maintain a clear bowel to allow for retraining (Flemming, 2020). Long-term retraining of the colon will require a gradual reduction of pharmacological therapy. Removal of fecal impaction in children can be achieved with oral or rectally administered pharmacological agents. Studies have shown that children prefer oral medications over rectal ones (Tabbers et al., 2014). PEG is the most studied potential oral medication for the removal of fecal impaction (Sood, 2021a) (Table 2). PEG is well tolerated by children and has minimal adverse effects. For example, a 1.0–1.5 g/kg/day dose for up to 6 days effectively removes fecal impaction in up to 95% of chronically constipated children (Youssef et al., 2002). Other oral medications used to remove fecal impaction in children are mineral oil, lactulose, sorbitol, magnesium hydroxide, magnesium citrate, bisacodyl, and senna. Stimulant laxatives (bisacodyl) combined with osmotic laxatives have been used in children as a standard of care but only as a rescue medication (Bonilla et al., 2020). In addition, children <11 years old can use bisacodyl only under pediatrician guidance (NHS, 2021). However, its safety profile, especially long-term use, is not well studied in children. For example, one retrospective study reported that bisacodyl is effective and safe to use in children for an extended period (Bonilla et al., 2020). The authors determined that about 57% of children (0.9–21 years of age) responded to bisacodyl treatment. The duration of use ranged from 1 to 77 months. Less than 1% of children had side effects.

Underlying physical causes should be considered when treating infants with chronic constipation. Hirschsprung disease, cystic fibrosis, and anorectal malformations should be assessed. It appears that among laxatives, PEG is the most studied medicine (Gordon et al., 2016). It can be replaced by the milk of magnesia, mineral oil, bisacodyl, or senna (Philichi, 2018). According to some investigators, the type of laxative is less important than careful adherence to the regimen, supporting a clear colon for a long enough time to allow retraining to occur (Sood, 2021a). The maintenance regimen should be continued for at least 6 months once regular bowel habits have been established.

### 4.2 Complementary treatment of constipation in children

About one-third of parents prefer to use natural approaches to relieve constipation in their children (Vlieger et al., 2008). In infants, it has been recommended to try temporary dietary interventions such as increased intake of juice from fruits high in non-digestible sorbitol, such as prune, pear, and apple, which have been mentioned above. The mechanism of action of this intervention is that an addition to the diet of a large amount of slowly absorbed or non-absorbable carbohydrates results in an increase in the water content in stool and the frequency of defecation (Gryboski, 1966; Kneepkens, 1989). However, large amounts of fruit juice can lead to diarrhea in infants (Heyman and Abrams, 2017).

**FIGURE 2:**
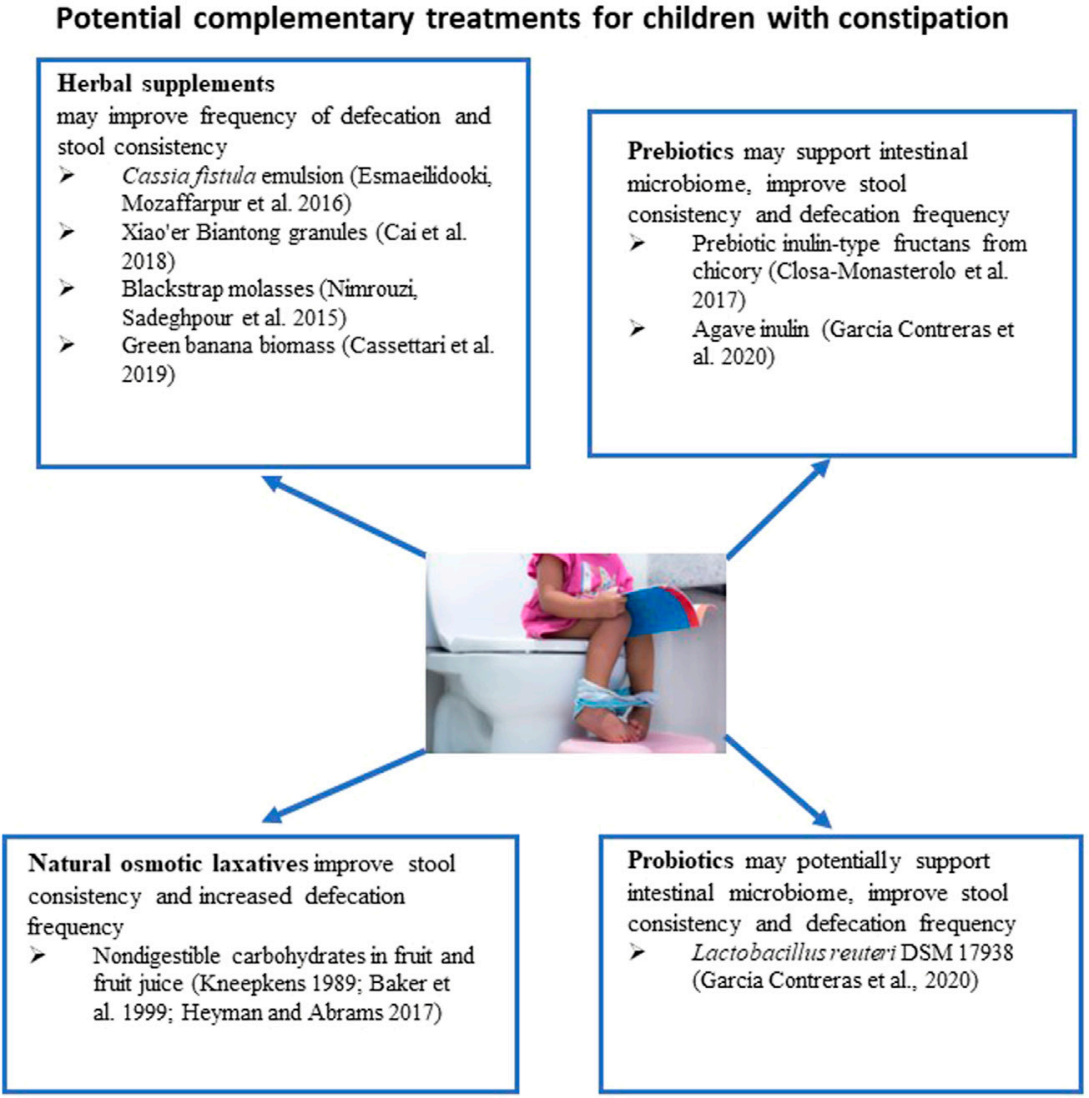
Potential Complementary Treatments for children with constipation.

The recommendation to use fruit juices and sorbitol to treat constipation in infants appeared in the 1999 NASPGHAN guidelines (Baker et al., 1999) (Figure 2). The guidelines noted that fruit juice might be a part of a dietary modification to maintain a clear bowel in constipated infants after the removal of fecal impaction. Other sources of slowly absorbed or non-absorbable carbohydrates mentioned included corn syrup and barley malt extract. The 1999 NASPGHAN guidelines highlight a lack of randomized, placebo-controlled trials investigating the efficacy of increasing fluid intake, supplemental fiber, or ingesting non-absorbable carbohydrates in pediatric patients (Baker et al., 1999). Interestingly, the 2014 NASPGHAN guidelines for constipation treatment did not address using non-absorbable carbohydrates from fruit juice or other sources as a dietary modification to treat constipation in infants (Tabbers et al., 2014). This could be due to the continued absence of randomized controlled trials investigating non-absorbable carbohydrates in constipated children. According to the guidelines, the available evidence for the dietary intervention did not support their recommendation for acute constipation (Tabbers et al., 2014). Still, a diet modification with fruit and fruit juice is a common clinical strategy to address acute constipation primarily because it is thought to be a condition resulting from the liquid-to-solid diet transition. In infants under 4 months of age, 1–2 ounces of 1:1 prune juice and water are suggested, while infants over 4 months of age should receive 2–4 ounces of prune, pear, or apple juice (Sood, 2021b).

It is recommended to increase the amount of food enriched with fiber in the diet of children older than 1 year suffering from acute constipation Sood, (2021b) Fiber is thought to retain water in its structure and improves the conditions for bacterial growth, thereby softening stools by increasing their weight and size (Stephen and Cummings, 1980). Fiber supplements considered safe for children and available without a prescription include psyllium, wheat dextrin, or methylcellulose. Additionally, care should be taken with children exhibiting withholding behaviors on fiber supplements, as this can exacerbate the potential for fecal impaction (Tabbers et al., 2014). In addition, a study investigated the effect of a mixture of fiber on the rate of defecation and stool pattern in children with chronic constipation previously treated with low-dose laxatives (Weber et al., 2014). While slightly higher rates of defecation and softer stools were reported in the fiber group compared to the placebo group, there was no significant difference between the two groups when comparing treatment failure rates, which was around 35%. In the analysis of four randomized, controlled trials of fiber efficacy in children with chronic constipation, the researchers could not determine if the fiber was effective in improving symptoms of constipation in children with chronic constipation (Tabbers and Benninga, 2015). Their work highlights the difficulty with which providers recommend fiber supplementation to children with chronic constipation. While some studies suggest favorable effects, the inconsistency of the definition of constipation, methodology, and outcomes as well as the small sample size, leave the body of evidence in a position of weakness (Tabbers and Benninga, 2015; Axelrod and Saps, 2018).

A recent systematic review analyzed the efficacy and safety of several non-pharmacological interventions, such as gut microbiome intervention and herbal remedies, in children with functional constipation (Wegh et al., 2022) (Figure 2). One study described in the review compared the efficacy of *Cassia fistula* emulsion with mineral oil in constipated children (Mozaffarpur et al., 2012). It is speculated that *Cassia fistula* acts like the anthraquinone laxative senna (Hakiminia et al., 2022) It has been found that the *Cassia* emulsion increased the defecation rate from 1.7 per week to 10.6 per week and was more effective in decreasing pain severity during defecation and stool consistency than mineral oil. In the subsequent study, the efficacy of *Cassia fistula* was compared to PEG for treating constipation in children (Esmaeilidooki et al., 2016) (Figure 2). It has been concluded that the effectiveness of Cassia treatment was comparable with PEG treatment. Both treatments were able to improve frequencies of defecation, the severity of pain, and the consistency of stool. In these studies, diarrhea was the most common adverse effect, with an incidence of 25%–32% of participants. However, this adverse effect was dose-dependent, as a 25% decrease in dose resulted in the resolution of diarrhea.

The effect of Xiao’er Biantong granules, a traditional Chinese medicine made up of a blend of plants, was compared to a placebo in 480 children (Cai et al., 2018) (Figure 2). Statistically significant improvements in the frequency of defecation were observed in the treatment group. In another study, the effectiveness of flixweed (*Descurainia Sophia* (L.) Webb ex Prantl) was compared with PEG in 120 children (Nimrouzi et al., 2015). No significant improvement in defecation frequency was observed when compared with PEG. One-third of children reported not liking the taste of flixweed. The efficacy of green banana biomass was investigated in a randomized clinical study conducted on 80 children with constipation (Cassettari et al., 2019) (Figure 2). The green banana biomass treatment significantly decreased the formation of hard-to-pass stool (e.g., nuts-shaped or sausage-shaped but lumpy stools), improved straining, and reduced pain following defecation. However, the frequency of defecation was not changed.

The efficacy of blackstrap molasses, a byproduct of sugar cane’s refining process, in relieving symptoms of functional constipation in children was compared with the efficacy of PEG treatment (Dehghani et al., 2019) (Figure 2). Ninety-two children with two or more of the following symptoms were included in the study: less than 3 defecations per week, straining lumpy or hard stool, sensations of incomplete evacuation or anorectal obstruction. Patients received 1 ml/kg of either molasses syrup or PEG syrup for 1 month. Both treatments significantly increased the number of defecations per week and relieved constipation symptoms. It appears that all children receiving molasses had more than 2 defecations per week after 2 weeks of treatment, while 6.7% of children receiving PEG had only two defecations per week. Unfortunately, these differences were not statistically significant. Molasses and PEG had a significant impact on the large stool diameter. Practically all participants had large stool diameter at the beginning of the study in both study groups; 22% of children receiving molasses and 15.6% in PEG group had large stool diameter after 2 weeks of treatment.

Several oral supplements, including *Cassia fistula*, Xiao’er Biantong granules, and blackstrap molasses, showed promising results and merited further study (Wegh et al., 2021). However, the authors noted that the current use of products like Xiao’er Biantong granules could be problematic. However, variability in composition can lead to difficulty establishing and recommending a specific product and dose. This challenge is similarly recognized in the European Society of Neurogastroenterology & Motility guidelines on functional constipation in adults (Serra et al., 2020), which suggests that certain herbal mixtures may be effective but that no specific product or dose can be recommended.

Probiotics have been suggested as potentially beneficial in chronic constipation due to the observation that individuals with chronic constipation have a different compositional microbiome in their intestines compared to healthy individuals (Zoppi et al., 1998) (Figure 2). In the case of constipation, probiotics may influence gut motility through the gut microbiome, fermentation, and effects on the nervous and immune systems (Dimidi et al., 2020). Available clinical trials have struggled to establish an effective dose, length of treatment, or strain. A 2017 double-blind, randomized clinical trial conducted in children <5 years old reported no differences in spontaneous defecation (“treatment success” defined as three times per week) between groups receiving a *Lactobacillus casei rhamnosus,* Lcr35 strain, and placebo (Wojtyniak et al., 2017). Critical evaluation of 17 randomized controlled trials led to the conclusion that data presented in these clinical trials do not support probiotic use as a sole treatment or complementary therapy of functional constipation in children (Harris et al., 2019).

The use of prebiotics to restore functional gut microbiota has been tested in children with constipation. A pilot randomized, double-blind, placebo-controlled trial was conducted to evaluate the efficacy of inulin-type fructan prebiotic in 17 children with chronic constipation (Closa-Monasterolo et al., 2017). Children were assigned to placebo or prebiotic groups. Stool hardness was improved in the prebiotic group; additionally, stool frequency increased in children assigned to the treatment group by ∼1 stool per week. The differences in the frequency of defecation were not statistically significant between the prebiotic and placebo groups. In another study, 24 children with cerebral palsy and chronic constipation were randomly assigned into placebo, prebiotic (agave inulin), and probiotic (*L. reuteri* DSM 17938) treatment groups (García Contreras et al., 2020). Throughout treatment, stool consistency showed significant improvement in the prebiotic and probiotic groups compared to placebo. Defecation frequency was also significantly improved, with an average of additional two occurrences per week in the prebiotic group.

The complementary treatments of constipation in infants and children presented above demonstrated some promising evidence of their effectiveness and safety in children. However, many evaluated trials lack the characteristics that would allow a comprehensive analysis of the evidence for or against complementary treatment use in pediatric patients. However, the available trials investigating complementary and conventional approaches can contribute to a more well-informed clinical decision by healthcare practitioners for use in infants and children with constipation.

## 5 Irritable Bowel Syndrome (IBS)

IBS is a constellation of symptoms primarily characterized by abdominal pain and either diarrhea or constipation that cannot otherwise be explained (Rasquin et al., 2006). It affects an estimated 6%–14% of children and 22%–35.5% of adolescents (Hyams et al., 1996; Lake, 1999; Miele et al., 2004). In addition, children and adolescents diagnosed with IBS experience lower pain thresholds in the rectum and altered perception of pain location upon rectal contraction (Van Ginkel et al., 2001; Halac et al., 2010). In addition, adolescents with IBS are also reported to have increased anxiety and depression (Hyams et al., 1996). Therefore, treating IBS in children and adolescents is essential for improved quality of life, including mental, physical, and social wellbeing.

### 5.1 Conventional treatment of irritable bowel syndrome in children

The guiding principles of treatment in children with IBS include education and behavioral modifications to support and maintain normal gut function. In support of these treatment mainstays, conventional drugs address individual symptoms. Conventional treatments for diarrhea and constipation are detailed in Section 2, Section 3. Conventional treatment for abdominal pain associated with IBS includes multiple classes of drugs, including antidepressants.

In adults, antidepressants are routinely used to treat IBS symptoms (Brandt et al., 2009). Their use in children, however, has been poorly studied. Among the few studied antidepressants is the antidepressant amitriptyline. A double-blind, placebo-controlled study evaluated “amitriptyline”s effect on abdominal pain in 33 adolescent participants with IBS (Bahar et al., 2008). There was no significant difference in the levels of abdominal pain between the treatment and placebo groups. Similar results from a placebo-controlled study of amitriptyline in 90 children were reported (Saps et al., 2009). These results suggest that amitriptyline does not improve abdominal pain in pediatric patients with IBS.

A systematic review conducted by a group of researchers evaluated the effectiveness of antidepressants, H2-receptor antagonists, serotonin antagonists, 5-HT4 receptor agonists, antihistamines, antibiotics, hormones, and antispasmodics for abdominal pain (Martin et al., 2017) (Table 3). In general, there is a lack of evidence to support the routine use of conventional drugs in the treatment of abdominal pain in children with IBS (Martin et al., 2017; Devanarayana and Rajindrajith, 2018). Additional research is necessary to understand the differences between adult and pediatric populations and obtain disease-specific data (Devanarayana and Rajindrajith, 2018). Current evidence (see Section 2, Section 3) does support the use of conventional drugs to treat diarrhea and constipation symptoms in pediatric populations with IBS.

**TABLE 3:** Conventional and complementary treatments of IBS in Children.

Name of the drug/treatment	Mechanism of action	Effectiveness/adverse events
Amitriptyline	Neurotransmitter modulation	It did not reduce abdominal pain (Bahar et al., 2008; Saps et al., 2009)
Antidepressants, H2 receptor antagonists, serotonin antagonists, 5-HT4 receptor agonists, antihistamines, antibiotics, a hormone, and antispasmodics	Neurotransmitter modulation	Evidence does not support the recommendation of these drugs to treat abdominal pain in children with IBS (Martin et al., 2017)

### 5.2 Complementary medicine in irritable bowel syndrome in children

Several complementary medicines are used to treat abdominal pain associated with IBS. Among them are acupuncture, hypnotherapy, relaxation techniques, and various herbal remedies (Chiou and Nurko, 2010). Current pediatric studies include probiotics, ginger, peppermint oil, and fiber.

Probiotics support a balanced intestinal microbiome (Devanarayana and Rajindrajith, 2018). The proposed mechanism of action of probiotics is to help restore a healthy microbiome that affects the luminal environment of the intestines, mucosal, and epithelial function, and healthy immune response (Lee and Bak, 2011). Multiple probiotic strains have been tested for efficacy and tolerability in children with IBS and associated symptoms. *Lactobacillus reuteri* is the most studied. Four randomized pediatric clinical trials of *Lactobacillus reuteri* DSM 17938 have been reported since 2016. Weizman et al.(2016) reported results from a randomized, double-blinded, placebo-controlled trial of 101 children. Results included significant declines in abdominal pain frequency and intensity after 4 weeks in the *L. reuteri* treated group. However, there was no difference between the groups for other gastrointestinal symptoms. In another prospective, randomized, double-blind, placebo-controlled study, *Lactobacillus reuteri* DSM 17938 was assessed in 26 children in the treatment group and 29 in the placebo group for abdominal pain after two and 4 months (Jadrešin et al., 2017). The treatment group showed a significant decrease in pain severity after 2 months.

**FIGURE 3:**
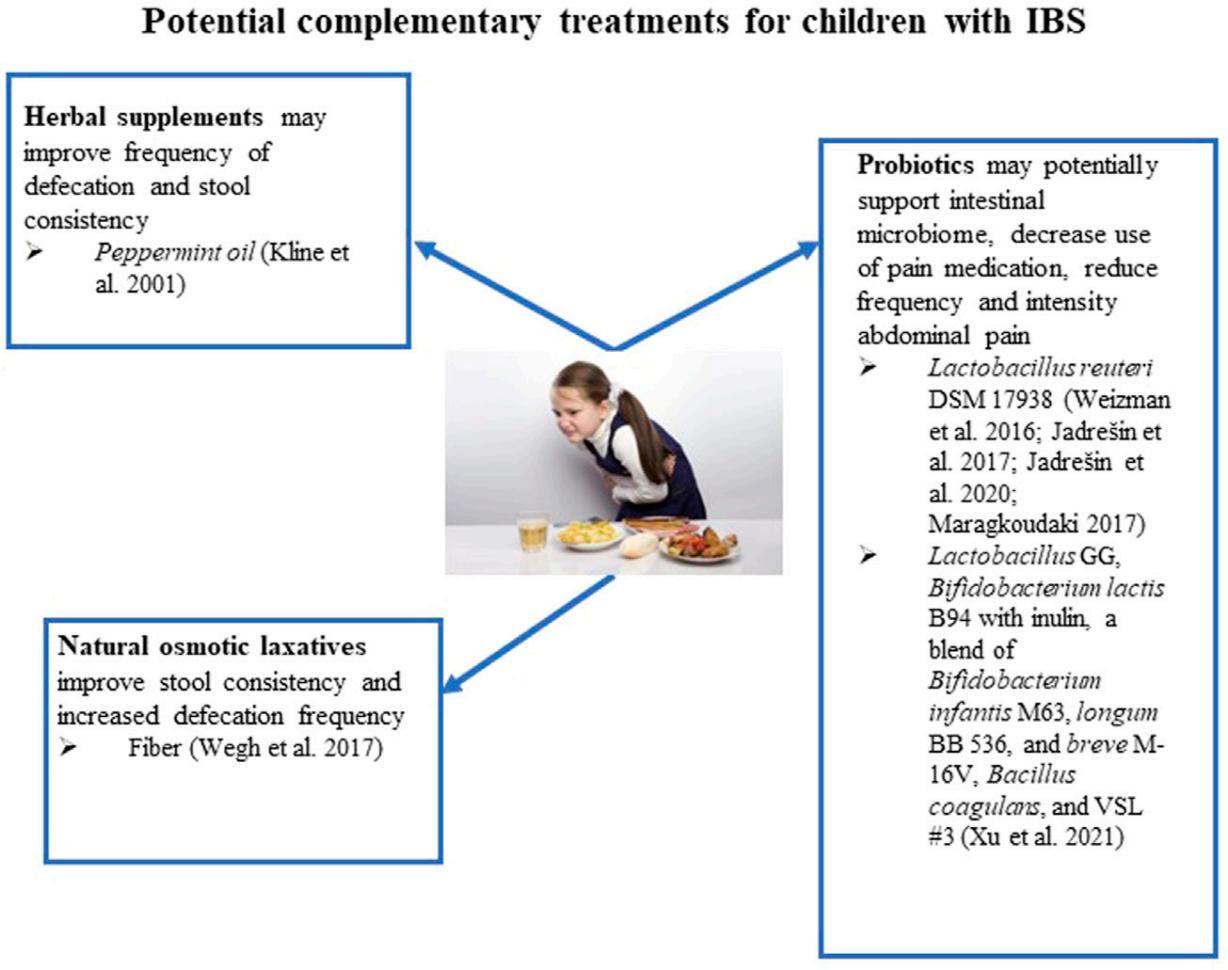
Potential complementary treatments for children with IBS.

Additionally, pain-free days were higher in the treatment group; however, after 4 months, a considerable reduction in abdominal pain was present in both groups. The authors of this study conducted a follow-up trial of 46 children over 12 weeks with a 4-week follow-up (Jadrešin et al., 2020). Abdominal pain severity decreased after 4 months in children treated with *Lactobacillus reuteri* DSM 17938. Pooled data with the previous trial revealed a consistent trend in the treatment group’s increased number of pain-free days. Comparing the groups when looking for children with complete cessation of symptoms revealed no significant difference (Jadrešin et al., 2020). Finally, Maragkoudaki et al. (2017) reported results from a study of 54 children randomized to either a strain of *Lactobacillus reuteri* DSM 17938 or a placebo for abdominal pain relief (Figure 3). The treatment and placebo groups reported a decrease in pain frequency and intensity after 4 weeks. Differences between treatment and placebo groups were not significant. Use of pain medication was reduced in the group treated with *Lactobacillus reuteri* DSM 17938 after 4 weeks (Maragkoudaki et al., 2017).

Other probiotic strains studied for use in children with IBS include *Lactobacillus* GG, *Bifidobacterium lactis* B94 with inulin, a blend of *Bifidobacterium infantis* M63, *Bifidobacterium longum* BB 536, and *Bifidobacterium breve* M-16V, *Bacillus coagulans*, and VSL #3 (mixture containing four strains of *Lactobacillus* (*Lactobacillus acidophilus*, *Lactobacillus plantarum*, *Lactobacillus casei*, and *Lactobacillus delbrueckii, bulgaricus*), three strains of *Bifidobacterium* (*Bifidobacterium breve*, *Bifidobacterium longum*, and *Bifidobacterium infantis*), and one strain of *Streptococcus* (*Streptococcus salivarius*, *thermophilus*). A recent systematic review and meta-analysis assessed randomized, placebo-controlled trials of these products (Xu et al., 2021). Participant inclusion criteria for the reviewed studies were enrollment of children between the ages of 4 and 18 that met the Rome II-IV diagnostic criteria for IBS. There was no restriction on the method of pain measurement, although treatment success was defined as an absence of pain. The reviewers also looked for patterns associated with the amount of daily administered probiotics and a standardized score of abdominal pain. Studies with repeated, poorly designed, animal, and incomplete data were not included in the systematic review. Nine trials met the inclusion criteria. Results indicated that the administration of probiotics in children with IBS significantly reduces abdominal pain. Probiotic administration also decreased the frequency of abdominal pain and increased the rate of successful treatment of abdominal pain (Xu et al., 2021). The reviewers found no meaningful relationship between the amount of daily administered probiotics and standardized abdominal pain.

In adults, multiple systematic reviews have illustrated the beneficial effect of orally administered peppermint oil on the symptoms of IBS. This benefit appears to be primarily due to peppermint’s spasmolytic effect in the gut *via* the modulation of calcium channels (Chumpitazi et al., 2018). This has led to a conditional recommendation by the American College of Gastroenterology guidelines for managing IBS (Alammar et al., 2019; Lacy et al., 2021). In children, however, the effect has been less studied. For example, a randomized, double-blind, placebo-controlled trial published in 2001 reported the results of enteric-coated peppermint oil capsules for treating IBS in children (Kline et al., 2001). In the two-center study, 50 children diagnosed by Rome or Manning criteria with IBS were enrolled. Exclusion criteria included children under 8 years old, children taking other medication for IBS or other medication that may affect abdominal symptoms, children weighing less than 30 kg, and children with other chronic conditions. The participants received 187 mg of peppermint oil (or 374 mg if over 45 kg) thrice daily for 2 weeks. Of the 50 enrolled participants, 42 completed the study. The participants that left the study had difficulty traveling to the study site, capsule swallowing difficulty, or concomitant administration of antibiotics. No adverse effects were reported. A significant reduction in pain severity was observed in 75% of the participants (Kline et al., 2001). It appears that peppermint oil had no effect on other IBS symptoms, such as heartburn, gas, belching, and stool consistency.

Supplementation with fiber has been suggested as a low-risk strategy for treating children with IBS accompanied by altered defecation patterns. By increasing stool bulk and intestinal gas from bacterial metabolic activity, stool regularity is thought to improve IBS symptoms (El-Salhy et al., 2017). A review in 2017 identified five randomized, placebo-controlled trials that assessed the effect of fiber supplementation on abdominal pain due to IBS in children (Wegh et al., 2017). It was noted that three trials reported a significant decrease in abdominal pain and number of episodes, while two trials reported no changes. This inconsistency in results, the reviewers wrote, could be due to methodological weaknesses in the two trials that saw no change in symptoms. Still, the variability in fiber type and amount tested, the primary outcomes, and the diagnosis of IBS were more challenging. Since no particular type of fiber or dose has been established in the available evidence, it is recommended to try daily psyllium or hydrophilic mucilloid fiber for about 4 weeks before assessing response (Chacko and Chiou, 2021). Generally, a total daily fiber intake target can be calculated by adding 5–10 g to the child’s age (Chacko and Chiou, 2021).

Limited trial data exist for conventional and complementary abdominal pain treatments in pediatric populations. However, many evaluated trials lack the IBS-specificity and power that would allow for comprehensive analysis. While additional analyses are necessary, existing data in adults and children indicate that complementary approaches, such as probiotics, peppermint oil, and fiber, may support conventional approaches and should inform clinical decisions.

## 6 Discussion

Significant improvements have been made in determining the effectiveness of complementary treatments for children with GID. Still, there remains enormous room for new rigorous studies pursuing unanswered questions and shoring up clinical results. Many existing studies lacked the sample size, methodological structure, or consistent measurements of successful treatments to do more than push the conversation in a productive direction, but this is still progress, however halting. A review of three GIDs and the state of the research can be well-summarized with the following general conclusions.

Pharmacological treatments of constipation face hurdles when it comes to definitive results. Currently, all pharmacological options for constipation are available only off-label and are non-approved by the FDA. Still, some pharmacological treatments are more well-researched than others; PEG, for example, has several studies upholding its effectiveness in reducing constipation and its associated symptoms (Youssef et al., 2002).

The available clinical studies support the efficacy of certain fruit juices, such as pear, prune, and apple juice, in lessening constipation symptoms. These foods are often found in the home, and their use can be considered a low-risk strategy for episodes of acute constipation. The potential relief from constipation comes from sorbitol in those drinks (Gryboski, 1966; Kneepkens, 1989). However, non-pharmacological treatments have found little definitive success in demonstrating efficacy against constipation (Tabbers et al., 2014). In particular, the body of research into probiotics for treating constipation is relatively thin and with conflicting results (Wojtyniak et al., 2017). Additionally, fiber, well-known as an essential tool for fighting constipation in adults, has no such clarity as a possibly effective complementary treatment for children (Weber et al., 2014). Small prebiotic trials have shown promising results, with one study we reviewed showing a statistically significant improvement in prebiotic patients compared to the placebo group (García Contreras et al., 2020).

Diarrhea claims more young lives by far than any other condition studied in-depth in this manuscript. The literature revealed some strong responses to multiple complementary treatments. Complimentary treatments that are most commonly used for diarrhea are simple oral rehydration therapy (King et al., 2003;Carson et al., 2016) (using restorative fluids containing high levels of electrolytes) and, in poorer countries, a zinc mineral supplement (Shane et al., 2017). Both have been found to mitigate diarrhea symptoms significantly. Additionally, the probiotic strain *S. boulardii* has produced moderately strong results in reducing the length of acute diarrhea (Szajewska et al., 2020b).

Research into the probiotic treatment of chronic diarrhea is less well-established than for acute diarrhea, but the limited results give some promising early indications of efficacy. A meta-analysis of studies into the zinc supplement’s effectiveness against chronic diarrhea found that, on average, it reduces the length of the condition by 16 h (Lazzerini and Wanzira, 2016).

Several probiotics have been tested for their effectiveness against children’s IBS-related symptoms, including abdominal pain. A meta-analysis of nine studies showed promising results in significantly reducing the severity and frequency of symptoms (Xu et al., 2021). No probiotic has been studied more for its effectiveness against pediatric IBS than *Lactobacillus reuteri.* It has shown promising results, although placebo groups also scored well on some pain reduction metrics after several months in some trials (Jadrešin et al., 2017). Meta-analysis research into fiber has also shown some preliminary promise, but with significant caveats that will necessitate the need for more research. These limitations include the studies’ large variability in thresholds for IBS diagnoses, the type of fiber administered, and definitions of successful outcomes (Wegh et al., 2017). Limited trial data evaluating conventional and complementary treatments for abdominal pain in pediatric populations lack the IBS-specificity and power that would allow for comprehensive analysis.

## 7 Conclusion

Providing the highest level of care to pediatric patients suffering from gastrointestinal conditions can be challenging. The evidence supporting the use of complementary treatments of GIDs in children is increasing in number and quality. However, more needs to be done to support the use of non-conventional therapies of GID in children. Sound clinical judgment is required to decide what conventional and complementary therapies may benefit a child suffering from a gastrointestinal condition/disease.

## Abbreviations

CDC, Centers for Disease Control and Prevention; ESPGHAN, European Society for Paediatric Gastroenterology, Hepatology, and Nutrition; FDA, Food and Drug Administration; GID, gastrointestinal disorders; GHHD, Gengen Huangqin Huanglian decoction; IBS, Irritable Bowel Syndrome; IDSA, Infectious Disease Society of America; NASPGHAN, North American Society for Pediatric Gastroenterology, Hepatology, and Nutrition; OTC, over the counter; PEG, polyethylene glycol; VILAR, All-Russian institute of Medicinal and Aromatic Plants; WHO, World Health Organization.
